# GERIATRICS IN HEALTH PROFESSIONS EDUCATION: A BIBLIOMETRIC ANALYSIS OF 70 YEARS OF RESEARCH

**DOI:** 10.1093/geroni/igad104.3370

**Published:** 2023-12-21

**Authors:** Renzo Pajuelo-Vasquez, Wendy Gutierrez-Baca, Javier Flores-Cohaila, Fernando M Runzer-Colmenares

**Affiliations:** Universidad Cientifica del Sur, Lima, Lima, Peru; Universidad Cientifica del Sur, Lima, Lima, Peru; Universidad Cientifica del Sur, Lima, Lima, Peru; Universidad Cientifica del Sur, Lima, Lima, Peru

## Abstract

To move forward in the field of Geriatrics in Health professions education (HPE), we must understand its conception and recognize current trends and future directions. Therefore, we conducted a bibliometric and network analysis of all studies indexed in the Scopus Database related to Geriatrics and HPE from 1953 to 2022. We retrieved 2940 studies comprising 7344 authors and 618 journals; these were imported to bibliometrics (RStudio) and Vos Viewer. To understand the intellectual structure, we employed a co-citation analysis; we identified 5 clusters that described fundamentals in geriatrics, nursing education, needs assessment, and interprofessional education (IPE). Furthermore, six topics were placed in the keyword co-occurrence, ranging from medical, nursing, dental, social workers, and interprofessional education. Consequently, we identified geriatrics education for medical students and interprofessional education between 2004 and 2016; furthermore, clinical competence was the most recent trending topic, accompanied by end-of-life care and geriatric psychiatry. In the evolution map, three topics emerged in the last ten years: IPE, online learning, and geriatric dentistry. These findings were confirmed in the bibliographic coupling analysis. Four major themes emerged among 13 clusters: IPE, Attitudes and career orientation to geriatrics, innovative educational methods such as gamification, and geriatric undergraduate education. Although we identified several studies, this barely represents the 1% of production in HPE. Furthermore, we identified a trend toward IPE, undergraduate medical education, career orientation, and innovative education. However, there is a lack of themes such as simulation and entrustable professional activities, with an underrepresentation of the developing world.

